# Post-Treatment HPV Surface Brushings and Risk of Relapse in Oropharyngeal Carcinoma

**DOI:** 10.3390/cancers12051069

**Published:** 2020-04-25

**Authors:** Barbara Kofler, Wegene Borena, Jozsef Dudas, Veronika Innerhofer, Daniel Dejaco, Teresa B Steinbichler, Gerlig Widmann, Dorothee von Laer, Herbert Riechelmann

**Affiliations:** 1 Department of Otorhinolaryngology, Medical University of Innsbruck, Anichstrasse 35, 6020 Innsbruck, Austria; jozsef.dudas@i-med.ac.at (J.D.); veronika.innerhofer@tirol-kliniken.at (V.I.); daniel.dejaco@i-med.ac.at (D.D.); teresa.steinbichler@i-med.ac.at (T.B.S.); herbert.riechelmann@i-med.ac.at (H.R.); 2 Division of Virology, Department of Hygiene, Microbiology, Social Medicine, Medical University of Innsbruck, Peter-Mayr-Strasse 4b, 6020 Innsbruck, Austria; Wegene.Borena@i-med.ac.at (W.B.); dorothee.von-Laer@i-med.ac.at (D.v.L.); 3 Department of Radiology, Medical University of Innsbruck, Anichstrasse 35, 6020 Innsbruck, Austria; gerlig.widmann@i-med.ac.at

**Keywords:** oropharyngeal squamous cell carcinoma, human papillomavirus, recurrence, surface brushing, EGFR

## Abstract

Human papillomavirus (HPV)-positive oropharyngeal squamous cell carcinoma (OPSCC) is a distinct subtype of head and neck cancer. Here, we investigated how frequently brushing remained high-risk (hr)-HPV positive after treatment and whether patients with positive post-treatment brushings have a higher recurrence rate. Following the end of treatment of patients with initially hr-HPV positive OPSCC, surface brushings from the previous tumor site were performed and tested for hr-HPV DNA. Of 62 patients with initially hr-HPV DNA-positive OPSCC, seven patients remained hr-HPV-DNA positive at post-treatment follow-up. Of the seven hr-HPV-positive patients at follow-up, five had a tumor relapse or tumor progression, of whom three died. The majority of patients (55/62) was HPV-negative following treatment. All HPV-negative patients remained free of disease (*p* = 0.0007). In this study, all patients with recurrence were hr-HPV-positive with the same genotype as that before treatment. In patients who were hr-HPV negative after treatment, no recurrence was observed.

## 1. Introduction

Approximately 85% of adults acquire a human papillomavirus (HPV) infection in their life. Most HPV infections are transient, asymptomatic, and eliminated by the immune system [1,2]. However, HPV viral infection can persist latently in a subset of the population. Individuals with persistent high-risk HPV (hr-HPV) infection may acquire epithelial cell abnormalities and subsequently develop cancers at the site of infection [3]. Persistent hr-HPV infection is particularly associated with cervical, anogenital and oropharyngeal cancers [4,5]. Hr-HPV positive oropharyngeal squamous cell carcinoma (OPSCC) is a distinct subtype of head and neck carcinoma. Risk factors that may prevent the natural clearance of oropharyngeal hr-HPV infection are genetic and lifestyle factors like smoking and alcohol consumption [6]. The prevalence of cancer of the oropharynx due to hr-HPV infection has increased, particularly in North America and Europe [7]. It differs from HPV-negative head and neck squamous cell carcinoma (HNSCC) by its risk factor profile, clinical behavior, and molecular biology. Compared to HPV-negative HNSCC, hr-HPV positive OPSCC better responds to treatment and has a significantly better prognosis [8]. 

It is unclear whether hr-HPV persists in oropharyngeal tissues in patients with hr-HPV-positive OPSCC following cancer treatment and which consequences this might have. Zhang and coworkers collected blood at diagnosis and post-treatment in 64 patients with p16-positive OPSCC to test for serum antibodies to E6 and E7 proteins of HPV 16. At diagnosis, most patients were seropositive to HPV 16 E6 (85%). In the post therapeutic samples, HPV 16 antibody levels decreased slowly over time, but only three patients became seronegative [9]. In another study, salivary and serum immunoglobulin G (IgG) antibodies targeting E2, E6, and E7 were measured in 44 patients with OPSCC at the beginning and 6–7 weeks following the completion of treatment. In this study, E7-directed antibodies were detected in saliva in most of the patients and were associated with the HPV status. The median of salivary E7 antibody levels decreased significantly post-treatment [10]. 

Fakhry and coauthors used oral rinse samples for HPV detection in 396 patients with oral and oropharyngeal cancer, of which 51% were HPV-positive before therapy. After treatment the HPV prevalence decreased. In patients who received surgical resection, the HPV prevalence decreased from 69.2% to 13.7%. In a subset of patients who required postoperative radiotherapy, the HPV prevalence decreased from 70% to 38% after surgery and then to 1% after radiotherapy. HPV detection in oral rinses was performed several times for patients who received radiotherapy. The median time to clearance was 42 days (95%CI, 37–49 days). The only factor significantly associated with reduced clearance was current smoking. HPV-positivity with the same genotype was detected after treatment in 14.3% of initially HPV-positive patients and among these patients, the cumulative incidence of recurrence was 45.3%. HPV DNA detection after completion of therapy was significantly associated with increased risk of recurrence and death [11]. 

In cervical dysplasia, hr-HPV infection can persist following treatment and promote disease recurrence. Söderlund-Strand et al. performed a long-term follow-up study obtaining cervical samples for HPV DNA testing and cytological analysis from 178 women with abnormal smears referred for conization. Three years after treatment 3.1% of women were persistently HPV-positive with the same HPV genotype as before treatment. Recurrent or residual cervical intraepithelial neoplasia (CIN) in histopathology was found among 9 (5.1%) women during follow-up. All these women had a type-specific HPV persistence. The authors concluded that only type-specific HPV persistence predicted recurrent or residual disease [12].

In a previous publication, we reported that surface brushings from oropharyngeal cancer reliably detect HPV-DNA. In 53 patients with OPSCC, sensitivity and specificity of the brush test was 86% (95%CI: 70–95%) and 89% (95%CI: 65–99%) [13]. Also, Broglie et al. reported liquid-based brush cytology specimens from oropharyngeal lesions to be a reliable method to identify patients with hr-HPV OPSCC. The authors collected brush cytology specimens prospectively from 50 patients with OPSCC. The accuracy, sensitivity, specificity, positive predictive value, and negative predictive value of brush cytology to identify hr-HPV-DNA-positive and p16-positive OPSCC samples were 88%, 83%, 94%, 95%, and 81%, respectively [14].

In this study, we examined whether hr-HPV is still detectable in surface brushings after treatment in patients with initially hr-HPV-positive OPSCC. Moreover, we compared the course of disease in patients with and without post-treatment hr-HPV in oropharyngeal brushings.

## 2. Results

### 2.1. Study Population and Treatment Outcome

During the study period, 74 patients with hr-HPV DNA-positive OPSCC before treatment were included. From 12 patients, no post-treatment brushings were available because they missed the follow-up or received follow-up in another hospital. The mean age of the remaining 62 patients with post-treatment brushings was 61 years. Forty-eight patients were male, and 14 were female. Most patients had UICC stage IVa and were treated with radiochemotherapy (Table 1). After treatment, all patients (except three) were in complete remission. One patient developed early pulmonal metastasis with complete remission at the primary site, another patient was in partial remission, and the third patient had already disseminated disease during initial diagnosis with pulmonal and bone metastases. This patient received a palliative systemic therapy. The median follow-up time was 29.3 months (95% CI: 25–34 months). 

### 2.2. HPV DNA Detection before and after Treatment

Before treatment, the most common HPV subtype was HPV 16 (Table 2). In 60/62 pre-HPV+ patients, p16 immunohistochemistry was available. In 55/60 patients, p16 was positive, and in five patients, it was negative. In the five patients with p16-negative IHC, the HPV genotypes 16, 16, 18, 16 and 40 (multiple infection), and 62 and 82 (multiple infection) were detected by pre-treatment brushing. At follow-up, 7/62 (11.3%) brushings from the previous primary tumor site were hr-HPV DNA-positive. In five patients, the post-treatment HPV brush test showed the same genotype as before therapy, namely HPV 16 in three patients and HPV 33 and HPV 18 each in one patient. All patients with HPV positivity after therapy with the same genotype as before developed a recurrence or progressive disease. 

**Figure 1:**
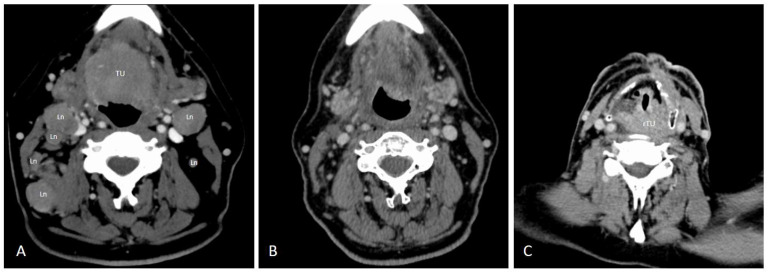
Patient presentation in radiological series. Ln, lymph nodes; TU, tumor; rTU, recurrent tumor. (**A**) Oropharyngeal cancer cT4cN2cM0. (**B**) Full remission after primary radiochemotherapy. (**C**) Recurrence involving the hypopharynx.

The post-treatment brushing was taken during complete response in one of the five patients positive with the same genotype. This patient with initially cT4cN2cM0 oropharyngeal cancer (Figure 1A) was disease-free after primary radiochemotherapy (Figure 1B) for 17 months and got a recurrence involving the hypopharynx three months after the post-treatment brush was obtained (Figure 1C). Another patient had a complete remission at the primary site but developed new pulmonary metastasis. The positive brushing was taken from normal oropharyngeal mucosa. The third patient had an early recurrence, and the post-treatment brushing was taken from the recurrent tumor surface and the fourth patient was in a palliative setting after diagnosis with a cT3cN2cM1 oropharyngeal cancer, he received four cycles of Carboplatin and Cetuximab and then Nivolumab 240mg/every two weeks. The fifth patient was diagnosed with an initially cT4cN2cM0 oropharyngeal cancer and was treated with primary radiochemotherapy. In this patient, partial remission 12 weeks and 18 weeks after therapy was found. The positive brush was taken from the surface of residual tumor. 

In two patients, post-treatment brushing was positive for another hr-HPV genotype than before therapy. The genotypes were HPV 16 and 33 before therapy and, in the same order of patients, HPV 68b and 16 thereafter. These two patients were in full remission after treatment, no recurrence or tumor persistence was stated in these patients. After treatment, one hr-negative patient was positive with the low-risk type HPV 6, and 54 patients were negative for all investigated HPV strains. 

### 2.3. Post-Treatment HPV-Positivity, Recurrent Disease, and Survival

In 4/7 patients with positive post-treatment hr-HPV DNA detection, a recurrence was observed, and in 1/7, tumor progression was observed (Table 3). In contrast, persistence or recurrence were observed in 0/55 patients who were post-treatment hr-HPV DNA-negative (*p* = 0.0007; OR 244.2; 95% CI: 10.4 to 5757.7). All post-treatment hr-HPV-positive patients were also p16-positive at initial diagnosis. Interestingly, all patients with recurrent or progressive tumor (5/62) were hr-HPV positive with the same genotype than before therapy. Three of these five patients died because of the recurrence or tumor progression 10, 12, and 35 months after diagnosis. In contrast, in the two hr-HPV positive patients after treatment without recurrence, another hr-HPV genotype was detected than before treatment. 

### 2.4. Factors Associated with Post-Treatment HPV-Positivity

**Figure 2:**
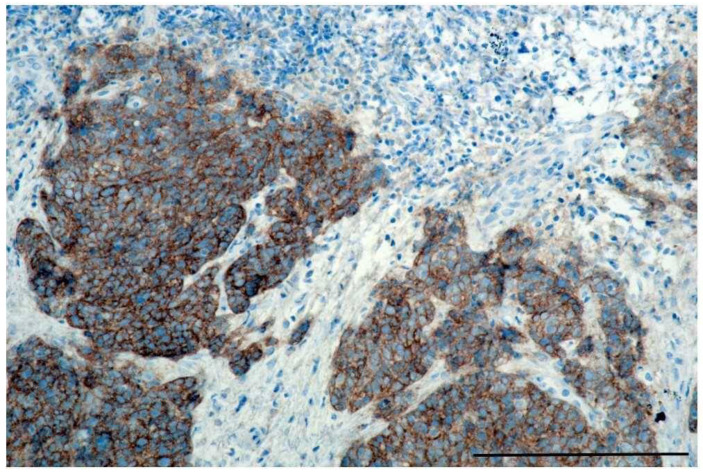
Epidermal growth factor receptor (EGFR) expression in a high-risk HPV (hr-HPV)-positive oropharyngeal squamous cell carcinoma (OPSCC) specimen. EGFR, epidermal growth factor receptor; hr, high risk; HPV, human papilloma virus, bar is 1 µm.

Post-treatment hr-HPV positivity was more frequent in patients whose primary tumor expressed epidermal growth factor receptor (EGFR) (Figure 2). EGFR expression was observed in 5/7 patients with and in 14/44 patients without post-treatment hr-HPV-positivity (*p* = 0.025). Another associated factor was advanced primary tumor T-stage (*p* = 0.031). No association was observed between post-treatment hr-HPV-positivity and UICC stage, treatment modality and radiotherapy dose. Post-treatment hr-HPV positivity was also not associated with PD-L1 expression, patient-reported smoking status, and consumption of alcoholic beverages at initial diagnosis. 

### 2.5. Post-Treatment HPV-Positivity Virus Persistence or New Infection?

Before therapy HPV 16 was the most common genotype (50 patients, 80.6%) and HPV 18 the second most common (4 patients, 2.5%). Other genotypes were HPV 33 (three patients, 1.9%) and HPV 35 (three patients, 1.9%). HPV 70, 66, 58, 82, 31, and 40 were detected each in a single patient or additionally to HPV 16 as a multiple infection. As mentioned before, after therapy five patients were hr-HPV-positive with the same genotype than before therapy. Only in these patients a recurrence or tumor progression was stated. The genotypes HPV16 were detected in three patients, and HPV 33 and HPV 18 were each detected in a single patient after therapy. As HPV 18 and HPV 33 are rare in the population of HPV-positive OPSCC patients, we suspect a virus persistence instead of a new infection. 

## 3. Discussion

In this study, we questioned whether patients with hr-HPV DNA-positive OPSCC remain hr-HPV DNA-positive after treatment and if post-treatment hr-HPV DNA at the initial tumor site is associated with the rate of disease persistence or recurrence. Before and after treatment, brushings were taken from the oropharynx, including the surface of the previous tumor site and tested for hr-HPV-DNA. Post-treatment brushings were available in 62 patients. Overall, 88.7% of hr-HPV-positive patients were hr-HPV negative at follow-up. In seven patients, hr-HPV after treatment was detected, and all patients hr-HPV-positive for the same genotype developed a recurrence or tumor persistence. Detection of hr-HPV at follow-up was associated with a substantially increased risk for persistent or recurrent disease (OR 244.2; 95% CI: 10.4 to 5757.7).

Post-treatment hr-HPV positivity and persistent or recurrent disease are rare events in hr-HPV-related oropharyngeal carcinoma. Accordingly, the results on potential influencing factors are based on a low number of patients and should be considered with caution. However, our results are in line with previous data. 

Also, Hanna and coworkers described a significant decrease in post-treatment E7 antibody levels in the salivary glands of patients with OPSCC [10]. Rettig and coworkers investigated hr-HPV DNA in oral rinses in 157 patients with OPSCC. At initial diagnosis, HPV type 16 was detected in 67/124 patients. After therapy, oral HPV 16 DNA was detected in six patients (9%). All five patients with persistent oral HPV 16 DNA developed a recurrent disease. Of these patients, three died. Persistent HPV 16 DNA detection in oral rinses was associated with a greater than 20-fold increased risk of recurrence (hazard ratio [HR], 29.7 [95% CI, 9.0–98.2]) and death (HR, 23.5 [95% CI, 4.7–116.9]) [15]. In a similarly designed study on 93 patients with OPSCC and HPV 16-positive cancer of unknown primary, pre- and post-treatment serum or saliva samples were taken to detect HPV 16 E6. The authors reported hr-HPV-positive post-treatment saliva to be associated with higher risk of recurrence (hazard ratio [HR], 10.7; 95% CI, 2.36–48.50) and reduced overall survival (HR, 25.9; 95% CI: 3.23–208.00). The combined saliva and plasma post-treatment HPV 16 DNA status was 90.7% specific and 69.5% sensitive in predicting recurrence within three years [16]. Fakhry and coauthors used oral rinse samples for HPV detection and described a significant decrease in HPV DNA after therapy, about 14.3% patients remained HPV-positive compared to 11.3% in our study. Also in this study, the authors described a significantly lower two-year overall survival among HPV-positive patients with persistent HPV detection for the same genotype (tumor-type) after therapy than among those without detectable tumor-type DNA after therapy (68% vs. 95%; adjusted HR, 6.61; 95% CI, 1.86–23.44; *p*  =  0.003), as was recurrence-free survival (55% vs. 88%; adjusted HR, 3.72; 95% CI, 1.71–8.09; *p*  <  0.001) [11]. 

Although, only few studies analyzed HPV-positivity after treatment in OPSCC patients and although the number of patients in our study is low, post-treatment HPV-positivity seems to be a strong predictor for overall survival. An inclusion of this observation in the clinical management in patients diagnosed with hr-HPV-positive OPSCC should be discussed. Post-treatment HPV detection in the early follow-up period can be a possible new standard to evaluate and predict the clinical course of those patients. As the brush test does not require biopsies and is easy to perform in awake patients without anesthesia, this is a very suitable and simple test method for HPV-DNA detection. Post-treatment hr-HPV-positive patients may require a very close clinical and radiological follow-up, these patients can be at high risk for a poor overall survival. One of our patients with post-treatment HPV-positivity received an HPV vaccination with Gardasil^®^9 (Recombinant Human Papillomavirus 9-valent Vaccine, Merck Sharp & Dohme BV, Haarlem, Netherlands) during full remission, hoping to reduce virus activity. However, he developed a locoregional recurrence. Interestingly, 2/7 patients were HPV-positive after therapy with a different hr-HPV genotype, these two patients were in full remission and experienced no recurrence. It seems that a new hr-HPV infection occurred and the risk in these patients for recurrence after therapy is as low as in post-treatment HPV-negative patients. However, higher numbers of patient are needed for further evaluation. 

Also, in patients with cervical cancer, persistent HPV infection is associated with an increased risk for recurrence [17]. In a study on 72 women with CIN, persistence or clearance of hr-HPV DNA was described as an early valid prognostic marker of failure or cure after treatment, more accurate than cytology or section margin status at the time of conisation. The absence of hr-HPV DNA had a 100% negative predictive value [18]. Söderlund-Strand et al. described a type-specific HPV-persistence in women with a residual CIN. No recurrent or residual disease was detected in women with any other patterns of HPV positivity, e.g., type change or fluctuating positivity [12]. 

In our study HPV 16 was the most common genotype before therapy, other genotypes like HPV 18, 33, and 35 were rare. Two patients were pre- and post-treatment positive for comparatively rare HPV genotypes 18 and 33. As reinfection with these rare types is unlikely, we assume tumor virus persistence rather than new HPV infection. Our findings about pretreatment HPV genotypes are in line with other studies. Chatfield-Reed and coauthors reported that of 99 hr-HPV positive HNSCC patients, 75.6% were positive for HPV 16 and 3% for HPV 18. In this study, 16.2% were positive for the genotype HPV 33, which we detected in only 1.9% [19]. Fossum and coworkers reported in 166 OPSCC patients (77% hr-HPV positive), HPV 16 to be the predominating genotype (65%), followed by HPV 33 (17%), HPV 18 (2%), and HPV 31/35/56/59 in one patient each [20]. 

In this study, post-treatment hr-HPV positivity was associated with clinical T-stage at diagnosis and tumor EGFR expression. An association of post-treatment hr-HPV positivity and primary tumor EGFR expression has not yet been reported. However, this observation is based on very few patients. EGFR protein over-expression has been reported in 70–100% of HNSCC, but 46–72% of OPSCC [21,22,23]. Although the reason why HPV-positive tumors express less EGFR expression is currently unknown, smoking has been hypothesized to be a contributory factor [24].

We did not observe an association of smoking and post-treatment hr-HPV positivity. This might be due to the low number of patients. Active smoking was admitted by 8/45 patients. Kero et al. reported a correlation between persistent oral HPV infection and smoking in 131 men who were sampled by serial oral scrapings. Genotype-specific HPV persistence was detected in 18/129 men. The mean persistence time ranged from 6 months to 30.7 months. The authors concluded that most of the persisting oral infections in males were caused by HPV 16, and smoking increased the oral hr-HPV persistence [25]. Another assumed risk factor for HPV persistence is immunosuppression. In a cohort of 97 HIV/AIDS patients, a genotype-specific oral and oropharyngeal HPV persistence was described in 33.3% of patients, of which 13.3% were hr-HPV positive [26].

However, the most significant observations of this study are that the majority of patients are hr-HPV-negative after therapy and that post-treatment hr-HPV positive patients seem to have an increased risk of tumor persistence or recurrence.

Limitations of the study are that not all OPSCC patients were enrolled consecutively in the study. From 12 patients, no post-treatment brushings were available because they missed the follow-up or received follow-up in another hospital. This includes also patients who may have had a relapse. Moreover, several otorhinolaryngologists were involved in sampling, but all were instructed how to collect samples. In this study, the brushings were obtained in some patients under general anesthesia during restaging panendoscopy and in some patients awake during clinical follow-up. 

The brush test is a simple test method and does not require tumor biopsies for HPV-DNA detection, future clinical usefulness of this test includes an oropharyngeal HPV screening for populations at risk, e.g., immunosuppressed elderly population. Partners of HPV-positive OPSCC patients, who are often concerned about oral HPV transmission, can also be tested by oropharyngeal brushings. 

## 4. Material and Methods

The aim of the study was to determine how many patients with hr-HPV-DNA-positive oropharyngeal carcinoma before treatment have positive hr-HPV-DNA detection in the former tumor region after completing therapy. We were also interested in whether post-treatment hr-HPV-DNA detection is related to disease recurrence. 

This prospective study was conducted in accordance with the GCP guidelines. Written informed consent was obtained from each patient who agreed to participate in this study following detailed explanation of the procedural workflow. Prior to any patient enrolment, the study had been approved by the institutional board in charge, i.e., the ethics committee of the Medical University Innsbruck, Austria. The respective reference number was 1147/2018. The study was conducted in full accordance with the principles expressed in the Declaration of Helsinki.

### 4.1. Inclusion and Exclusion Criteria

Patients with incident oropharyngeal cancer positive for HPV-DNA before treatment were included. Virus DNA detection was performed by tumor surface brushings and/or by HPV-DNA isolation from formalin-fixed and paraffin-embedded (FFPE) tumor biopsies [13]. All patients were treated at the Department of Otorhinolaryngology—Head and Neck Surgery, Medical University of Innsbruck between April 2014 and October 2019 according to the recommendations of the interdisciplinary tumor board. Patients were invited to undergo a post-treatment surface brushing from the initial tumor site at least 12 weeks following end of treatment. Patients who were not able or willing to undergo a post-treatment brushing were excluded from the analysis. The follow-up for HNSCC patients is standardized, the restaging is performed 8–12 weeks after the treatment and includes a CT scan and panendoscopy with sampling from the former tumor region. In the case of full remission, further clinical and radiological controls are arranged every six weeks for three times, every three months for three times and then every six months. The follow-up ends five years after diagnosis.

### 4.2. Specimen Harvest and Handling

Before the start of the treatment, all patients underwent routine endoscopy under anesthesia, including tracheobronchoscopy, esophagoscopy, and laryngopharyngoscopy. During this examination, a cytology brush (Digene^®^ HC2 DNA Collection Device, Qiagen, Hilden, Germany) of the tumor surface was taken. The brush test reliably detects HPV-DNA in patients with OPSCC, sensitivity and specificity of this method is 86% (95%CI: 70–95%) and 89% (95%CI: 65–99%) [13]. The brush was then kept in a sterile container in 0.05% sodium azide and sent to the Division of Virology of the Medical University of Innsbruck. Biopsies of the tumor were then obtained, fixed in formalin, and sent to the Department of Pathology for routine histopathological investigation. Moreover, immunohistochemistry (IHC) of p16, epidermal growth factor receptor (EGFR), and programmed death-ligand 1 (PD-L1) was performed. 

After completion of therapy, a second surface brushing was taken from all subsites of the oropharynx, including the initial tumor site. This second surface brush was obtained during follow-up examinations at least 12 weeks after therapy during a restaging endoscopy or during clinical follow-up. 

### 4.3. Processing of Brush Specimens

Nucleic acid extraction was performed within two days of arrival of the samples at the laboratory. The DNA extraction was performed in a fully automated manner (NucliSens^®^ easyMAG^®^, Biomerieux, Marcy-l’Étoile, France) [27,28]. A total of 500 µL of the sample was pipetted into a disposable well. After an initial cell lysis, the nucleic acid components were isolated from the mixture using magnetic silica particles, resulting in a total of 110 µL purified DNA extract. Approximately 5–10 µL of this extract was used for HPV-DNA detection and genotyping. 

### 4.4. DNA Amplification and HPV Genotyping

For the detection of HPV DNA, real-time polymerase chain reaction (PCR) was used based on the amplification of the L1 open reading frames (ORF). As an internal control for the availability of cell material, a PCR for the household beta-globin gene was performed in parallel. The HPV DNA was considered positive if the fluorescence signal appeared before the 40th cycle [29]. In the next step, all HPV-positive samples were further genotyped after an amplification step using reverse line blot hybridization on nitrocellulose membrane strips containing genotype-specific probes (AmpliQuality HPV-TYPE EXPRESS, AB Analitica^®^, Padova, Italy) [30]. The genotyping kit is able to identify 40 different HPV types, namely 6, 11, 16, 18, 26, 31, 33, 35, 39, 40, 42, 43, 44, 45, 51, 52, 53, 54, 55, 56, 58, 59, 61, 62, 64, 66, 67, 68a/b, 69, 70, 71, 72, 73, 81, 82, 83, 84, 87, 89, and 90 (AB Analitica^®^, Padova, Italy). Using the 2′-deoxyuridine 5′-triphosphate/uracil-DNA N-glycosylase dUTP/UNG system, which degrades non-specific residual RNAs, contamination due to carry-over was minimized. The detection limit of the test kit is 1000 viral copies per ml for HPV 16 and HPV 6, which is in accordance with the required detection limit by the World Health Organization (WHO) and the international HPV reference center [31]. Based on the International Agency for Research on Cancer classification, the HPV genotypes were classified as established high-risk, probably high-risk, established low-risk, and uncharacterized [32,33].

### 4.5. IHC

Five-micrometer-thick paraffin sections were dewaxed, and antigens were retrieved in an automated staining system (Ventana, Discovery, Tucson, AZ, USA). A commercial in vitro diagnostic (IVD) certified assay containing ready-to-use prediluted mouse monoclonal antibody was used for p16 detection (CINtec^®^ Histology V-Kit, Roche Diagnostics, Basel, Switzerland). EGFR was detected by a rabbit monoclonal IVD certified antibody (clone: A20-E) of Diagnostic Biosystems (Kosice, Slovakia), PD-L1 was detected by a rabbit monoclonal antibody (clone:E1L3N^®^) of Cell Signaling Technology (Frankfurt am Main, Germany), both of these primary rabbit monoclonal antibodies were used in a 1:200 final dilution in a Ventana Discovery staining system. Staining was completed using universal secondary antibody solution, hematoxilin counterstaining, and the DAB MAP Kit (all Ventana products, Ventana, Discovery, Tucson, AZ, USA). The tumor cell areas were evaluated by one experienced observer. Specimens were judged p16-positive if ≥60% of the cells in tumor areas revealed immunohistochemical reaction products. Staining of fibroblastic stroma cells was not counted. EGFR staining was scored based on previous publications as a positive membrane reaction in tumor cell nests: “0”: no staining, “1”: 1–9%, “2”: 10–49%, “3”: over 50% of the cells positive [34,35]. PD-L1 was estimated in cells located in tumor cell areas as follows: “0”: no staining, “1”: 1–5%, “2”: 5–10%, and “3”: at least 10% of the cell stained, as published by Ferris et al. in 2016 [36]. 

### 4.6. Data Analysis

Frequency data are tabulated and analyzed with Fisher’s exact test or with the Kruskal–Wallis test for ordered alternatives [37]. To calculate 95% CIs of proportions, the Wilson method with continuity correction was used [38]. For continuous data, means, and standard deviations (SD) are provided unless stated otherwise. Median follow-up was calculated by with the reverse Kaplan–Meier method [39].

## 5. Conclusions

In this study, most patients with initially hr-HPV-positive OPSCC were HPV-negative after treatment, none of these patients experienced a recurrence. The five patients with recurrence or tumor progression were all post-treatment hr-HPV-positive with the same genotype. No recurrence was observed in two post-treatment hr-HPV-positive patients with a different genotype than at diagnosis.

## Figures and Tables

**Table 1:** Study population.

Variation	Patients (*n* = 62)
Gender	
Male	48
Female	14
Age during Initial Diagnosis	
≤ 50	10
51–60	21
61–70	20
71–80	7
>80	4
UICC Stage	
stage I	0
stage II	5
Stage III	18
stage IVa	32
stage IVb	4
Stage IVc	2
ASA Score	
ASA I/II	52
ASA III/IV	9
Therapy	
Surgery only	3
Surgery and PORT	12
Surgery and RCT/RIT	5
Primary RCT/RIT	38
Primary RT	2
Chemotherapy only	1
P16	
Negative	5
Positive	55

ASA, American Society of Anesthesiologists; UICC, Union internationale contre le cancer; PORT, postoperative radiatiotherapy; RCT, radiochemotherapy; RIT, radioimmunotherapy; RT, radiotherapy.

**Table 2:** Human papillomavirus (HPV) genotypes before and after therapy.

Patient Number	HPV Genotype before Therapy	HPV Genotype after Therapy	Recurrent Tumor/Tumor Progression
1–50	single hr-HPV type *	HPV negative	No
51–55	multiple hr-HPV types **	HPV negative	No
56–58	hr-HPV 16	hr-HPV 16	Yes
59	hr-HPV 33	hr-HPV 33	Yes
60	hr-HP 18	Hr-HPV 18	Yes
61	hr-HPV 33	hr-HPV 16	No
62	hr-HPV 18	hr-HPV 68b	No

HPV, human papilloma virus; hr, high risk; * single hr-HPV infection with one of the genotypes HPV 16, 33, 35, or 58; **multiple hr-HPV infections, including almost 1 hr-HPV genotype.

**Table 3:** Patients with post-treatment HPV-positivity for the same genotype.

Patient 1–5	UICC	Age at Diagnosis	ASA Score	HPV Pre-Treatment	Therapy	HPV Post-Treatment	Course of Disease
Patient 1	Stage III	55	2	HPV 16	RCT	HPV 16	Recurrence
Patient 2	Stage I	54	2	HPV 16	Surgery and PORT	HPV 16	Pulmonal metastasis Locoregional control
Patient 3	Stage III	73	3	HPV 16	RCT	HPV 16	Recurrence
Patient 4	Stage IV	86	2	HPV 33	CT only	HPV 33	Tumor progression
Patient 5	Stage III	70	2	HPV 18	RCT	HPV 18	Recurrence after partial response

HPV, human papilloma virus; ASA, American Society of Anesthesiologists; UICC, Union internationale contre le cancer; PORT postoperative radiotherapy; RCT radiochemotherapy; CT, chemotherapy.
